# European healthcare professionals’ use and experience with aripiprazole once-monthly 400mg two-injection start initiation regimen in adult patients with schizophrenia

**DOI:** 10.1192/j.eurpsy.2025.2108

**Published:** 2025-08-26

**Authors:** M. Yildirim, C. Beckham, A. Fagiolini, K. Leopold, W. J. Cottam, J. Hickey, O. Rogerson, S. Pappa

**Affiliations:** 1 H. Lundbeck A/S, Valby, Denmark; 2 Otsuka Pharmaceutical Europe Ltd., Berkshire, United Kingdom; 3 Università Di Siena, Siena, Italy; 4 Department of Psychiatry, Psychotherapy and Psychosomatics, Vivantes Hospital am Urban and Vivantes Hospital im Friedrichshain, Charite, Universitätsmedizin, Berlin; 5 Department of Psychiatry and Psychotherapy, Carl Gustav Carus University Hospital, Technische Universität Dresden, Dresden, Germany; 6 Real-World Evidence, OPEN Health; 7 Division of Psychiatry, Department of Brain Sciences, Imperial College London, London, United Kingdom

## Abstract

**Introduction:**

Aripiprazole once monthly 400mg (AOM400) is a long-acting injectable (LAI) available with a two-injection start initiation regimen (AOM400-TIS) for the maintenance treatment of adult patients with schizophrenia stabilised with oral aripiprazole.

**Objectives:**

This survey sought to explore the perspectives and experiences of HCPs using AOM400-TIS across Europe.

**Methods:**

HCPs who had prescribed and/or administered the AOM400-TIS regimen to ≥3 patients with schizophrenia were invited to participate in an online survey. The survey was launched in two waves across the target countries (wave 1: Italy, Germany, United Kingdom (UK); wave 2: Denmark, Italy, Sweden). The survey aimed to understand HCPs’ perspectives and attitudes towards prescribing and/or administering AOM400-TIS according to the European label in clinical practice, including reasons for its use, potential benefits, and common barriers and/or concerns. Analysis was descriptive; data was collected between February 1–March 21, 2024 (wave 1) and September 16-October 28 (wave 2).

**Results:**

216 HCPs completed the survey (wave 1: 31 from Italy, 31 Germany, 32 UK; wave 2: 28 from Denmark, 64 Italy and 30 Sweden) including psychiatrists (67%) and psychiatric nurses (27%). HCPs estimated 30.0% (median; IQR: 20.0–50.0) of patients in their caseload were diagnosed with schizophrenia, and of these, 50.0% were treated with LAIs (median; IQR: 25.0–65.0). 46% of HCPs were primarily responsible for prescribing AOM400-TIS, 26% for administering it, and 28% were responsible for both. HCPs estimated that 42% of patients typically spent 14–28 days on oral aripiprazole prior to AOM400-TIS, with HCPs rating the severity of symptoms of patients initiated with AOM400-TIS as mild (18% of HCPs), moderate (65% of HCPs) and/or severe (53% of HCPs). The most common reasons for initiating AOM400-TIS after transitioning from oral aripiprazole were poor adherence (88%) and relapse(s) (52%), and the most reported goals for prescribing AOM400-TIS were to improve adherence (69%) and prevent relapses (64%). Common barriers to the use of AOM400-TIS were patient reluctance to receive two injections (55%), concerns about tolerability (34%) and safety of administering a high dose in a single day (35%). Prior treatment adherence (56%) and efficacy (46%) were the most cited factors influencing prescribing of AOM400-TIS. Overall, HCPs “agreed”, or “strongly agreed”, that AOM400-TIS was easy to administer (84%) and that it had a similar safety/tolerability profile to the single injection start regimen (60%), while the majority were satisfied with patient outcomes with AOM400-TIS (85%).

**Conclusions:**

Overall, HCPs with experience of using AOM400-TIS reported that it is easy to administer, well tolerated, and improves treatment outcomes, while barriers to its use include patient reluctance and perceived safety concerns.

**Disclosure of Interest:**

M. Yildirim Employee of: Murat Yildirim is a full time employee of H.Lundbeck A/S, Valby, Copenhagen., C. Beckham Employee of: Clodagh Beckham is a full-time employee of Otsuka Pharmaceutical Europe Ltd., Berkshire, UK., A. Fagiolini Grant / Research support from: Angelini, Boehringer Ingelheim, Lundbeck, Janssen, Otsuka, Pfizer, Recordati, Viatris, Consultant of: Angelini, Boehringer Ingelheim, Idorsia, Italfarmaco, Lundbeck, Janssen, Medicamenta, Mylan, Otsuka, Pfizer, Recordati, Rovi, Sunovion, Teva, Viatris, K. Leopold Grant / Research support from: Janssen, Otsuka, Consultant of: Boehringer Ingelheim, Lundbeck, Janssen, Otsuka Recordati, ROVI, Speakers bureau of: Boehringer Ingelheim, Lundbeck, Janssen, Otsuka Recordati, ROVI, W. Cottam Employee of: Will Cottam is a full-time employee of OPEN Health (London)., J. Hickey Employee of: Joe Hickey is a full-time employee of OPEN Health (London)., O. Rogerson Employee of: Olivia Rogerson is a full-time employee of OPEN Health (London)., S. Pappa Grant / Research support from: Recordati, Janssen, Consultant of: Lundbeck, Janssen, Otsuka, Recordati, Rovi, Gedeon Richter, Sunovion, Speakers bureau of: Lundbeck, Janssen, Otsuka, Recordati, Rovi, Gedeon Richter, Sunovion.

